# Inhibition of cell-adhesion protein DPYSL3 promotes metastasis of lung cancer

**DOI:** 10.1186/s12931-018-0740-0

**Published:** 2018-03-07

**Authors:** Yang Yang, Yan Jiang, Dong Xie, Ming Liu, Nan Song, Junjie Zhu, Jiang Fan, Chenfang Zhu

**Affiliations:** 1 Department of Thoracic Surgery, Shanghai Pulmonary Hospital affiliated Tongji University, 507 Zhengmin road, Shanghai, 200433 China; 2 Department of General Surgery, Shanghai Ninth People’s Hospital, Shanghai JiaoTong University School of Medicine, Discipline Construction Research Center of China Hospital Development Institute, Shanghai Jiao Tong University, 639 Zhizaoju road, Shanghai, 200011 China

**Keywords:** DPYSL3, LLC cells, EMT, Lung cancer, Metastasis

## Abstract

**Background:**

Our previous screening study suggested that the cell-adhesions protein Dihydropyrimidinase-like 3 (DPYSL3) was a candidate metastatic lung cancer related molecule. This study aimed to analyze the correlation between DPYSL3 and metastatic lung cancer.

**Methods:**

Stable DPYSL3 knockdown Lewis lung carcinoma (LLC) cells were constructed with a retroviral system. Cell migration and invasion assays were performed to determine the role of DPYSL3 in LLC cells’ migration and invasion changes. A metastatic lung tumor model in which the stable DPYSL3 knockdown LLC cells were injected through tail vein was used to analyze the role of DPYSL3 in tumor metastasis in vivo. The correlation between DPYSL3 expression and the survival time of lung cancer patients were analyzed in KMPLOT database.

**Results:**

Knockdown of DPYSL3 promoted the migratory and invasive of LLC cells compared to the control group. Meanwhile, the motility of LLC cells was also increased with the inhibition of DPYSL3. The TGFβ-induced EMT increased when DPYSL3 was inhibited. The expression of EMT markers, TWIST1 and N-cadherin, significantly increased to almost two times with the knockdown of DPYSL3. Furthermore, inhibition of DPYSL3 promoted the progression of metastatic xenograft in C57BL/6 mice. The expression level of DPYSL3 decreased in lung cancer patients with distant metastasis.

**Conclusions:**

Knockdown of DPYSL3 promoted the metastatic ability of LLC cells in vitro and in vivo.

## Background

Lung cancer is the leading cause of cancer mortality around the world [1]. Although the advanced improvements have been made in the diagnosis and treatment, the 5-year survival rate of lung cancer patients is still far from satisfactory. One of the main reasons is that the metastasis emerges. Metastasis is a dynamic interaction between cancer cells and microenvironments. It was thought as a late event and only occurred when primary lesion had progressed locally. However, recent evidences suggested that metastasis also occurred in early-stage due to the spread of circulating tumor cells [2, 3].

Although several pathways, including the Wnt/β-catenin pathway and the transforming growth factor-β (TGFβ) pathway, have been identified promoting the metastasis of cancer cells [4], further scientific research is required to elaborate the process.

In order to analyze the lung cancer metastasis related components, we performed a screening assay and found that the dihydropyrimidinase Like 3 (DPYSL3) might contribute to the occurrence of metastasis in lung cancer.

DPYSL3, also named as CRMP4, is a member of cytosolic phosphoproteins family. It mediates the semaphorin/collapsin-induced growth cone collapse and regulates the neuronal differentiation [5, 6]. The previous research about DPYSL3 mainly focused on development of nervous system as well as its related disease. Recently, different DPYSLs family members had been confirmed to be related with the metastasis of colon and prostate tumors [7, 8]. Therefore it aroused our interest to ask what was the role of DPYSL3 in the metastasis of lung cancer. In this study, we observed the effect of DPYSL3 on cell motility, migration and invasion of lung cancer cells and analyzed the role of it in xenograft model. Furthermore, we confirmed that the increased TGFβ-induced epithelial-mesenchymal transition (EMT) caused by knockdown DPYSL3 might be responsible for metastasis of lung cancer.

## Methods

### Ethical approval

The protocol of this study was conducted according to the revised Declaration of Helsinki and approved by the Institutional Review Board (IRB) of Shanghai Pulmonary Hospital (Tongji University). Informed consent was obtained for experimentation with all participants. The privacy rights of participants were observed. All animal experiments were carried out in accordance with the National Institutes of Health guide for the care and use of Laboratory animals (NIH Publications No. 8023, revised 1978).

### Reagents and antibodies

Lipofectamine 2000 (Life Technologies), TGFβ (R&D Systems) and rabbit antibodies against TWIST (CST #46702), N-Cadherin (CST #4061) and GAPDH (CST #2118), were purchased from the indicated manufacturers. LLC cells were obtained from ATCC and cultured in DMEM supplemented with 10% FBS. To induce the EMT, the LLC cells were treated with 2 ng /mL TGFβ for 48 h.

### Constructs

Mammalian expression plasmids for DPYSL3 were constructed following the recommendation of molecular cloning. The DPYSL3-RNAi target sequence were: #1, TACATGGAGGATGGCTTAATA; #2, CACCACCATGATCATTGACCA. The knockdown efficiency of DPYSL3-RNAi was determined at both mRNA and protein level.

### Cell motility assay

Cell motility was assessed through scratch wound assay. Monolayer cells were seeded in six-well cell culture plates for 24 h. Then, the 200 μl pipette tip was used to introduce the wound with a width of 400–600 μm. Subsequently free-floating cells and debris were removed through PBS washing. The retained cells were cultured with serum-free medium. The wound healing was observed and photoed by the microscope 36 h later.

### Stable DPYSL3 knockdown LLC cells

The lentiviral production was used to construct the stable cell lines.

The viral was produced with the HEK293 cells which were transfected with packaging plasmids together with the DPYSL3-RNAi plasmid. Twenty-four hours later, the medium was changed into DMEM medium without antibiotics and the cells were incubated for another 24 h. Then the LLC cells were transfected with the virus. They were cultured with the filtered recombinant virus-containing medium for 24 h and selected with puromycin (0.5 μg/ml). The control cells, which were stable GFPi knockdown LLC cells, were constructed with the same protocol. The knockdown efficiency was determined at both mRNA and protein level.

### Cell migration and invasion assay

The 12-well transwell plates (8.0 μm pore filters) (Corning, NY) were used to determine the invasion and migration of LLC cells. For cell migration assay, the LLC cells (1 × 10^5^) were seeded in the upper chamber of the transwell plate with serum-free DMEM medium. The DMEM medium with 10% FBS was added into the bottom chamber. The plate was incubated at 37 °C for 24 h. Then, the membrane of transwell was took off, fixed with 95% ethanol and stained with crystal violet staining. The cells got through the membrane were calculated under the microscope. For cell invasion assay, firstly, the membrane of 12-well transwell plate was coated with matrigel. Then the LLC cells were seeded in the upper chamber. The following protocol was the same with the migration assay.

### Immunoblotting analysis

Cells were digested with 0.5% trypsinase and lysed with NP-40 lysis buffer on ice. Cancer tissues were put into homogenizer to grind into tissue homogenate with NP-40 lysis buffer. The concentration of each sample was determined through commercial kit. Then, the targeted molecules were fractionated through SDS/PAGE electrophoresis and subsequently transferred into the PVDF membrane. After blocking with 5% skimmed milk, the blot was incubated with the indicated antibody for 1 h. Following PBST washing, the blot was incubated with the appropriate secondary antibody. Then, it was developed with the ECL developing solution.

### Metastatic lung cancer models

The 8-week-old male athymic immunodeficient Balb/c C57BL/6 mice were purchased from Shanghai laboratory animal center. The stable DPYSL3 or GFPi knockdown LLC (2 X 10^7^) cells were injected via tail vein under strict aseptic operation. Lung tissues were harvested 6 weeks later and fixed in 4% PFA for histological characterization. The HE staining was performed according to the manufacturer’s instruction.

### qPCR

Total RNA was isolated and purified from cancer tissues with Trizol (Invitrogen, Gaithersburg, MD, USA). It was transcripted into cDNA with the Invitrogen commercial kit. The details of RNA amplification method and subsequent enzymatic reaction have been previously reported [9].

Total RNA was isolated for real-time PCR analysis to measure mRNA levels of the indicated genes. RNA was extracted (NIH purified usingedA was isolated for real-n,me PCR analysis to measure mRNA levels of the indicated genes. RNA was USA) following the manufacturer’s instructions. qRTPCR was performed on a Real-Time PCR system with SYBR Green qPCR Mix (Bio-rad, Hercules, CA, USA). For each gene, at least three separate sets of qRT-PCR analyses were performed. Data shown are the relative abundance of the indicated mRNA normalized to that of GAPDH.

### Statistical analysis

All data were analyzed by the SPSS package for Windows (Version 18.0, Chicago, IL). t test was used to analyze the results of cell motility, proliferation, migration and invasion assay. The statistically significant refers to the *P* value < 0.05.

## Results

### Knockdown of DPYSL3 promotes the motility of LLC cells

**Fig. 1:**
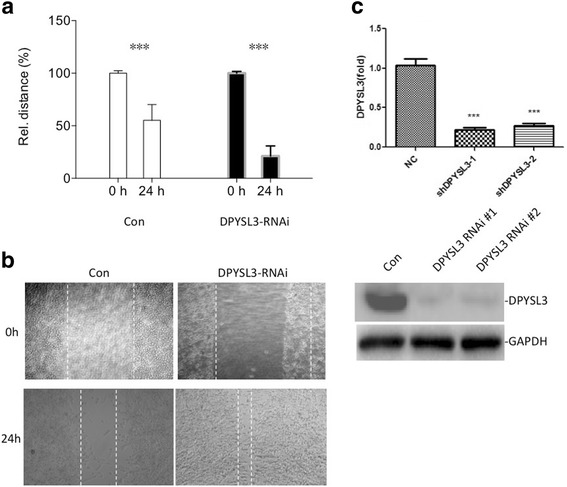
The motility of LLC cells increased when DPYSL3 was inhibited. **a** & **b** The wound closed faster with the knockdown of DPYSL3. **c** The knockdown efficiency of the two DPYSL3-RNAi used in this study. ***: *P* < 0.01

**Fig. 2:**
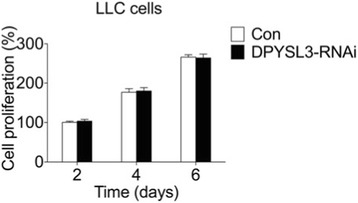
The proliferation of LLC cells was not affected by DPYSL3. Compared to the control group, knockdown of DPYSL3 did not influence the LLC cells’ growth at day 2, 4 and 6

In order to further analyze the role of DPYSL3 in progression and invasion of lung cancer, we first observed the motility changes of LLC cells caused by knockdown DPYSL3. As shown in Fig. 1, inhibition of endogeneous DPYSL3 significantly promoted the motility of LLC cells. Then we investigated the effect of DPYSL3 on cell proliferation, our data suggested that knockdown of DPYSL3 did not influence the growth of LLC cells (Fig. 2).

### Knockdown of DPYSL3 increases the migration and invasion of LLC cells

**Fig. 3:**
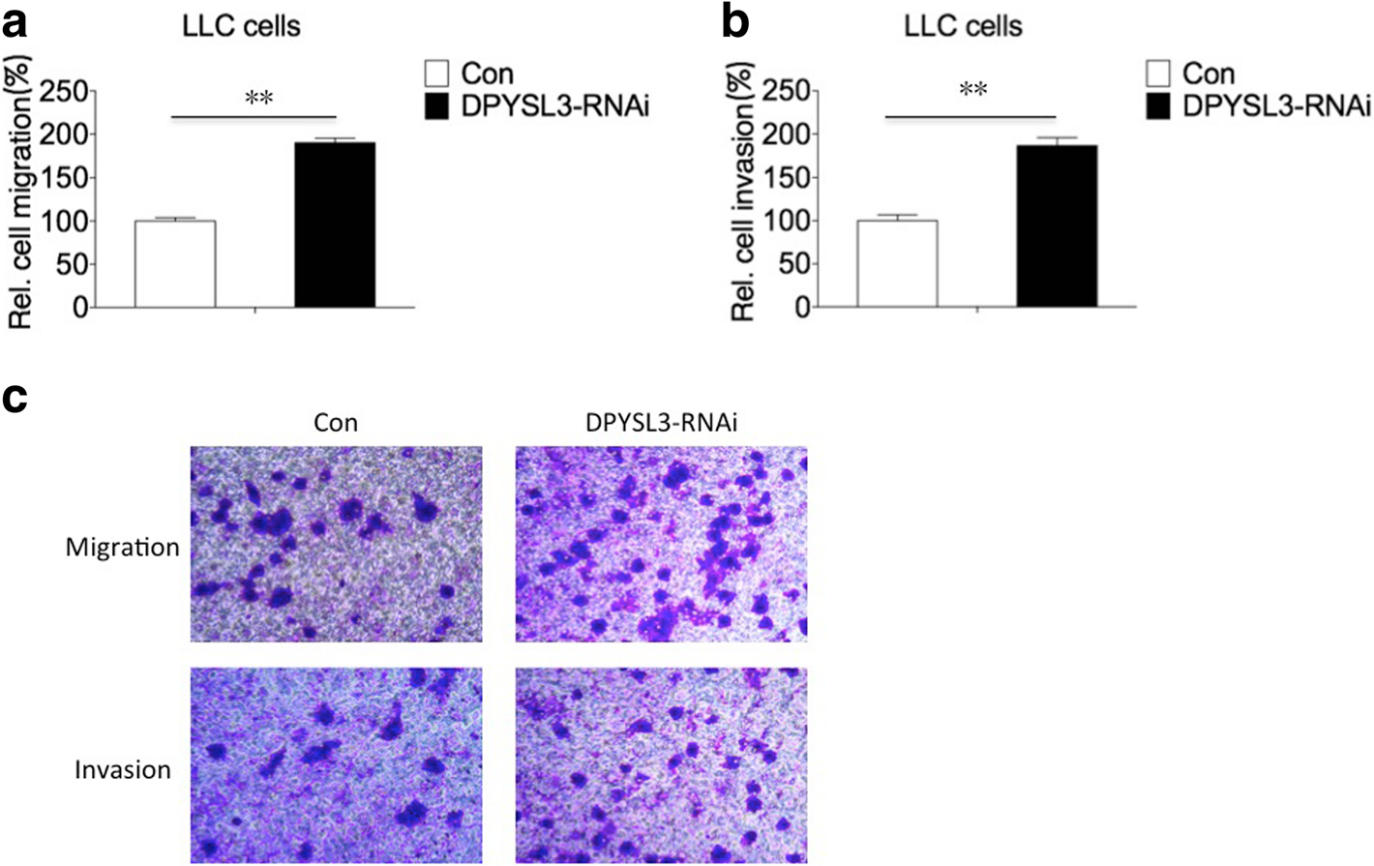
DPYSL3 influenced the migration and invasion of LLC cells. **a** Inhibition of DPYSL3 promoted the migratory ability of LLC cells. **b** The invasive ability of LLC cells increased with the knockdown of DPYSL3. **c** The original picture of migratory and invasive transwell assay. **: *P* < 0.01

The excessive migration and invasion are critical characters of tumor cells. We analyzed the migration and invasion changes of LLC cells with the inhibition of DPYSL3. As shown in Fig. 3, knockdown of DPYSL3 increased the migration and invasion of LLC cells. There were around 60% more cells passed through the transwell membrane compared to the control group (*P* < 0.01).

### Knockdown of DPYSL3 promotes TGFβ-induced EMT

**Fig. 4:**
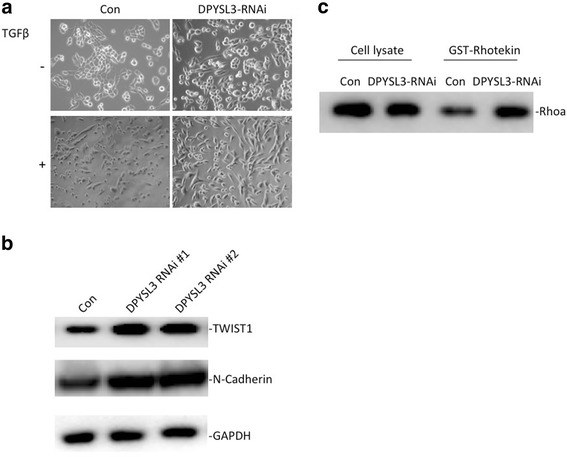
The TGFβ-induced EMT was affected by DPYSL3. **a** Decreased DPYSL3 induced the up-regulation of EMT. The LLC cells were treated with TGFβ (2 ng /mL) for 48 h. **b** The expression of EMT markers, TWIST1 and N-cadherin, increased when DPYSL3 was inhibited. **c** The RhoA activity also increased with the knockdown of DPYSL3

Accumulating evidence confirmed that EMT played a key role in cancer progression. Therefore, we further investigated whether DPYSL3 affected this process. When DPYSL3 was knockdown, the morphology of the LLC cells greatly changed into spindle and fibroblast-like shape (Fig. 4a). The expression of EMT markers, TWIST1 and N-cadherin, also increased when DPYSL3 was inhibited (Fig. 4b). Furthermore, the activity of RhoA, which promoted the metastasis of lung cancer, increased with the knockdown of DPYSL3 (Fig. 4c).

### Knockdown of DPYSL3 potentiates metastasis in mouse xenograft model

**Fig. 5:**
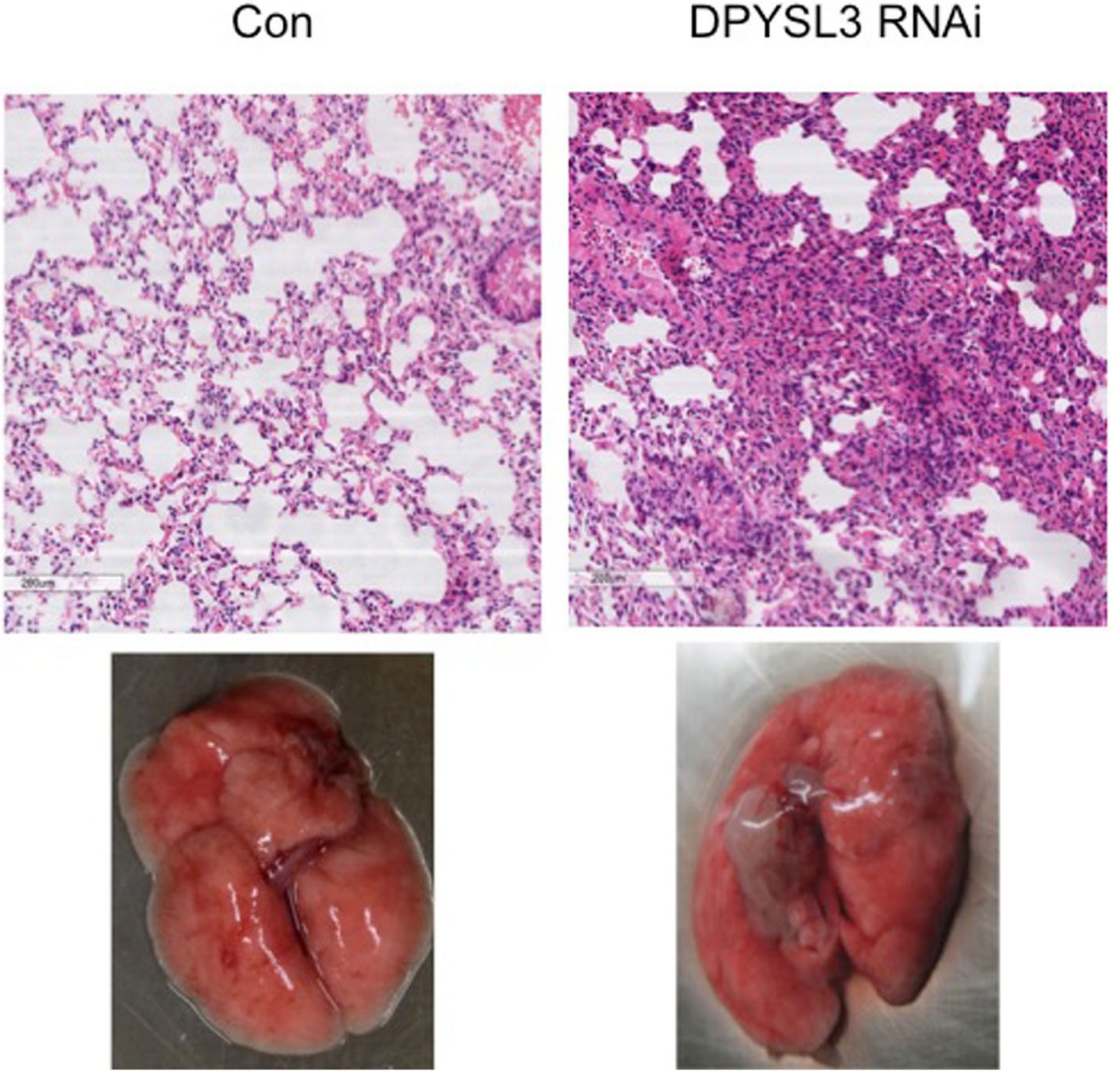
Knockdown of DPYSL3 potentiated the metastasis of LLC cells in vivo. There were more ulcers or tubercles in DPYSL3 knockdown group compared to control group 6 weeks post-injection

After we confirmed that inhibition of DPYSL3 promoted the EMT of LLC cells, we observed the effect of knockdown DPYSL3 in metastasis in vivo. Inhibition of DPYSL3 promoted the progression of metastasis in C57BL/6 mice. DPYSL3 stable knockdown LLC cells induced more ulcers compared to the control group. The HE staining also confirmed the results (Fig. 5).

### The expression of DPYSL3 decreased in lung cancer patients with distant metastasis

**Fig. 6:**
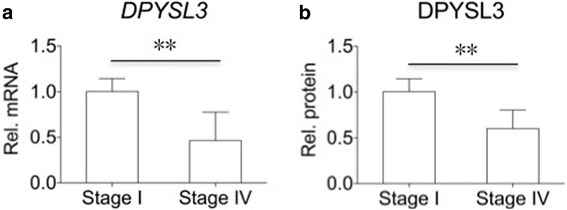
The expression of DPYSL3 decreased in lung cancer patients with distant metastasis. Compared to patients with stage I lung cancer (*N* = 10), patients with advanced lung cancer (stage IV) (*N* = 10) had higher expression of DPYSL3 at both mRNA (**a**) and protein (**b**) level. **: *P* < 0.01

Next, we analyzed the expression level of DPYSL3 in lung cancer patients. Compared to patients with localized lesion (stage I) (*N* = 10), patients with distant metastasis (stage IV) (*N* = 10) had lower expression of DPYSL3 at both mRNA and protein level (Fig. 6).

## Discussion

Metastasis is a major cause of cancer related mortality of lung cancer. However, the mechanism underlying the progression of lung cancer metastasis is still poorly understood and the specific treatment target is still missing. The absolute damage caused by the distant metastasis in lung cancer exceeds that of local lesion. With the help of improved biological knowledge, several metastasis suppressors including kinases and GTP-binding proteins have been identified [6, 10]. While the mechanism underlying their correlation is still unknown.

Our previous screening assay indicated that DPYSL3 might be a candidate metastatic lung cancer related molecule. The members of DPYSLs family were closely related to cancer progression and invasion. DPYSL1 was considered to be a lung cancer invasion suppressor gene [11]. DPYSL2 had been identified as a colorectal carcinoma biomarker [12]. It was also a negative regulator of p53 and had oncogenic activity [13, 14].

DPYSL3, which is a cell-adhesion protein, has been reported to be involved in the metastasis of tumors [4, 15–17]. But it played the opposite role in different cancers. In pancreatic cancer, inhibition of DPYSL3 reduced cellular invasion [14, 18]. While in prostate cancer, overexpression of DPYSL3 decreased the cellular invasion and inhibited tumor metastasis [4, 6, 15]. The correlation between DPYSL3 and lung cancer metastasis was still unknown. Therefore, in this study, we further analyzed the role of DPYSL3 in lung cancer metastasis. Our results suggested that DPYSL3 had influence on motility, migration and invasion of lung cancer cells. Furthermore, the occurrence of metastasis was inversely associated with the expression level of DPYSL3 in lung cancer patients. In order to elaborate the underlying molecular mechanisms of how DPYSL3 regulated the metastasis of lung cancer, we focused on the EMT changes and confirmed that knockdown DPYSL3 promoted TGFβ-induced EMT in LLC cells.

## Conclusion

In this study, we confirmed that DPYSL3 regulated the metastasis of lung cancer. DPYSL3 is involved in several signaling pathways [19–21] and the agonist of it is sufficient to have therapeutic efficacy. Therefore it might be a candidate treatment target to prevent the metastasis of lung cancer.

## Abbreviations

DPYSL3: Dihydropyrimidinase-like 3
EMT: Epithelial-mesenchymal transition
LLC: Lewis lung carcinoma
TGFβ: Transforming growth factor-β
